# The Book World of Medicine and Science

**Published:** 1903-02-21

**Authors:** 


### The Book World of Medicine and Science.

The Prize Essay on the Erection of a Sanatorium for the Treatment of Tuberculosis in England. By Arthur Latham, M.A. Oxon., M.A. Cantab., in association with A. William West, Architect. (London: Baillière, Tindall and Cox. 5s. net.)

This essay was submitted in competition for the prize offered by the Advisory Committee of the King's Sanatorium in January 1902.

In response to the advertisement, which was addressed to medical men of all nationalities, 180 essays with plans were submitted, of which the one before us was placed first by the Advisory Committee.

We looked forward to the publication of a Prize Essay with considerable interest, expecting to find in it a storehouse of up-to-date knowledge and some valuable practical suggestions as to the planning and construction of the buildings. The result, we must confess, is disappointing. The Essay itself is well put together and presented in an attractive form. The information got together if not, apparently, based on real experience, is at any rate copious, and many sources have been tapped in search of it. When, however, the authors come to what is, after all, the most essential part of their work—the practical application of their theories—their inadequate equipment for the task they have set themselves is but too apparent. The plans have the appearance of being the work of an amateur with a certain knowledge of the use of a T-square and scale, but who has very rudimentary ideas of planning.

In the instructions issued by the committee it was laid down that the sanatorium was to accommodate 100 patients, 50 of each sex, and that of this total number 88 were to be for the more necessitous class, whilst 12 were to be reserved for the well-to-do. In the block plan of the buildings which the authors recommend, the main administration buildings and the buildings for patients are grouped together in five separate blocks. The centre block is for administration; to the east is a block for 36 female patients, and another of 16 patients who are confined to bed. To the west is a block for 36 male patients, and a block for the 12 paying patients. Whether or not there is any great advantage to be gained by housing patients in detached blocks is open to question, but undoubtedly the system is difficult and also more expensive to supervise. What strikes us as particularly remarkable in the present plan is that the block for patients who are confined to bed, and who would therefore require most attention, is the one which is placed furthest from the administration block.

The blocks themselves are planned with two wings diverging at an angle of 145°, connected together by an open verandah which looks due south. One block therefore has a S.S.W. aspect; the other block a S.S.E. aspect. This is an arrangement good in itself as tending to secure the greatest amount of available sunshine, and also protection from wind.

Each wing contains 18 separate bedrooms for patients on two floors. Each bedroom has a square projecting window, 7 feet long and 4 feet deep. Thus there will be a recess about 4 feet square between the projecting window of each bedroom. The object of this recess is stated by the authors to lend space for a sofa chair. We question very much the advantage of this arrangement, while the impossibility of treating it at all successfully from an architectural point of view, is obvious.

The sanitary offices are placed at the extreme end of each wing. This arrangement appears to us an exceedingly bad one, inasmuch as it occupies valuable space which could have been devoted to open balconies. The argument used by the authors in defence of the arrangement is hardly convincing.

The administration building contains the dining-hall for the patients. The only access to it is apparently along one corridor off which the medical officers' rooms are placed, and a corresponding corridor on the other side where the nurses' room and committee-room are to be found. The matron is apparently put to guard one entrance, for her sitting-room, bedroom, with a w.c. opening out of the latter, and office, are all grouped together close to the recreation-hall on the ground floor. The kitchen offices are both badly arranged and quite inadequate. No separate provision appears to have been made for milk, and the larders open directly into the kitchen. Where the nurses have their meals is not apparent, as only one room is provided for them, which presumably is a sitting-room.

The detached building contains all that the authors appear to think necessary in the way of a laundry, boiler-house, etc. The laundry consists of two rooms, an ironing-room and a washing-house, both apparently of very small dimensions, and with the drying-horses in the ironing-room. No arrangements are made for sorting linen, either before or after washing, and no special provision is made for dealing with foul linen. Considerable space is given to research laboratories, and there is a large engine-room, but where the boilers are is not apparent.

An alternative plan is suggested—grouping the patients and the administration into one large block. In the plan this resembles to some extent the form of the separate blocks, but the very large projection in the centre to south, containing two libraries and a recreation-hall, is a fatal objection to this plan. Again certain of the offices are arranged round a central court at the back of the recreation hall, and the patients on their way to the dining-hall have to pass on the one side the matron's rooms and on the other side the secretary's rooms, and close to the dispensary and rooms for throat and dental purposes.

The suggestions on construction are many of them equally futile. The authors suggest that the floors should be made of steel girders laid on felt and filled in with concrete, the internal partitions being made of cement. This method of construction, they say, reduces the sound and is light. Now this method neither reduces sound nor is light. Nothing could be worse for its facility for conveying sound than a cement partition, and every architect knows the difficulty of deadening sound with a concrete and steel floor. Then, having advised that the floor should be of concrete and steel, they go on to say that the ordinary type of ceiling, with its dirt-accumulating space underneath joists, be not used. Of course it would not be used if the floor is made of solid concrete, and the argument for a zinc ceiling is beside the question altogether.

On the question of floor coverings, the authors recommend teak, and we are inclined to agree with them; but, if as some authorities hold, there should be no dry-rubbing whatever in a sanatorium, it is difficult to see where the particular advantage of teak comes in, because one great point about it is that when wax-polished it presents an absolutely impervious surface, and this surface must certainly be dry-cleaned and not washed.

After a careful perusal of this book, we are unable to see in what way, except perhaps by the information gathered together in the appendices, the advisory committee are to helped in arranging the sanatorium, and if this really be the best dissertation on the subject which was submitted to the committee, all we can say is that the rest must have been extremely bad.

